# Crystal structure of the essential biotin‐dependent carboxylase AccA3 from *Mycobacterium tuberculosis*

**DOI:** 10.1002/2211-5463.12212

**Published:** 2017-04-04

**Authors:** Matthew Bennett, Martin Högbom

**Affiliations:** ^1^ Department of Biochemistry and BiophysicsArrhenius Laboratories for Natural SciencesStockholm UniversitySweden

**Keywords:** drug design, FASII, lipid metabolism, Rv3285, tuberculosis, tyrosine

## Abstract

Biotin‐dependent acetyl‐CoA carboxylases catalyze the committed step in type II fatty acid biosynthesis, the main route for production of membrane phospholipids in bacteria, and are considered a key target for antibacterial drug discovery. Here we describe the first structure of AccA3, an essential component of the acetyl‐CoA carboxylase system in *Mycobacterium tuberculosis* (MTb). The structure, sequence comparisons, and modeling of ligand‐bound states reveal that the ATP cosubstrate‐binding site shows distinct differences compared to other bacterial and eukaryotic biotin carboxylases, including all human homologs. This suggests the possibility to design MTb AccA3 subtype‐specific inhibitors.

**Database:**

Coordinates and structure factors have been deposited in the Protein Data Bank with the accession number 5MLK.

**Abbreviations**
FAS: fatty acid synthase
MTb: *Mycobacterium tuberculosis*

*Mycobacterium tuberculosis* (MTb) is the causative agent of tuberculosis. Despite the existence of antibiotic treatments and a vaccine it remains one of the worst global killers with an estimated 1.5 million deaths and 10 million new cases yearly. There is a rapid development of drug‐resistant strains which pose a severe threat to TB control worldwide 1. Characterization of potential anti‐TB drug targets is therefore imperative. The bacterium has one of nature's most elaborate lipid metabolisms, which produces a complex and unique cell wall, that is also key to its virulence and inherent drug resistance. It is thus a primary drug target for current and future drugs 2, 3, 4, 5.

The type II fatty acid biosynthetic pathway is the main route for production of the acyl chain of membrane phospholipids in bacteria and is considered an excellent target for antibacterial drug discovery 6, 7. In MTb, the type II pathway works downstream of the type I fatty acid synthase (FAS) to produce very long‐chain lipids such as mycolic acids, an essential component of the mycobacterial cell envelope 6, 8, 9.

The biotin‐dependent Acetyl‐CoA Carboxylase catalyzes the regulated and committed step in the type II fatty acid biosynthesis, adding a carboxyl group to a coenzyme‐A ester, typically acetyl‐CoA 6, 10, 11. In mycobacteria, the enzyme complex is composed of two catalytic proteins AccA and AccD. AccA is a bifunctional protein with a C‐terminal biotin carboxyl carrier domain and an N‐terminal biotin carboxyltransferase domain catalyzing the reaction:

$$\text{ATP}+{\text{HCO}}_{3}^{-}+\text{AccA‐Biotin}\to \text{ADP}+P_{i}+{\text{AccA‐Biotin‐CO}}_{2}^{-}$$

The AccD transcarboxylase enzyme then transfers the CO_2_ from carboxybiotin to the CoA ester substrate 6, 12, 13.

The MTb genome encodes three annotated AccA homologs (AccA 1–3) and six annotated AccD homologs (AccD 1–6) 2, 14. The different AccD subunits confer specificity for the CoA‐lipid substrate 9, 11, 15. Despite the redundancy, transposon mutagenesis identifies no fewer than five of the genes (AccA2 and AccA3 as well as AccD4, AccD5, and AccD6) as individually essential for growth of the bacterium 16, 17.

The AccA and AccD proteins form large complexes, expected to contain six subunits of each protomer 11. MTb AccA3 has been shown to form functional Acetyl‐CoA Carboxylase complexes with AccD4, AccD5, and AccD6 14, 15, 18, 19, 20. The MTb AccA3‐AccD4 Acetyl‐CoA Carboxylase carboxylates long‐chain acyl‐CoA substrates. These are utilized in biosynthesis of mycobacterial specific very long‐chain fatty acids such as mycolic acids 11, 15, 18. AccA3–AccD5 have been shown to act mainly on propionyl‐CoA, producing methylmalonyl‐CoA, used for synthesis of methyl‐branched lipids, key components of the mycobacterial cell wall. AccA3–AccD6 acts mainly on acetyl‐CoA, producing malonyl‐CoA for fatty acid biosynthesis 11, 15, 21, 22.

*Mycobacterium tuberculosis* AccA3 thus provides the carboxylated biotin to all of the three essential AccD proteins 14, 15, 18, 19, 20. Moreover, the same interaction pattern has been found in MTb 11, 23. Together, this positions AccA3 as a protein of absolute importance for type II fatty acid biosynthesis in mycobacteria. Its direct involvement in mycolic acid synthesis and cell wall permeability further underlines the importance of AccA3 as a potential drug target 5, 8, 14, 18, 20, 23.

While structures have been determined for three of the MTb AccD proteins, AccD1 (PDB:4Q0G), AccD5 22, and AccD6 24, there is no structure available for any of the AccA proteins. Here, we present the 1.94 Å structure of a full‐length construct of MTb AccA3. As predicted from sequence, AccA3 adopts the three‐domain ATP‐grasp superfamily fold 25, 26. The protein crystallized as a dimer in the asymmetric unit with the monomers displaying different structural states, showing conformational dynamics between domains. The structure, sequence comparisons, and modeling of ligand‐bound states reveal that the biotin‐binding site is highly structurally conserved. The loop structure bridging the substrate‐binding sites and forming part of the ATP‐binding site, however, shows interesting differences compared to other bacterial and eukaryotic biotin carboxylases, suggesting the plausibility of designing MTb AccA3 subtype‐specific inhibitors.

## Materials and methods

MTb AccA3 (Gene name accA3, Rv3285 retrieved from Tuberculist (http://tuberculist.epfl.ch) 2) was produced from a synthetic gene, which was codon optimized for *Escherichia coli* expression (MWG, Ebersberg, Germany), and hosted in a modified pET28 plasmid which conferred a StrepII tag to the N terminus of the produced protein. AccD6 was expressed untagged from pETDUET (Novagen). Both proteins were produced separately in Rosetta II cells (Novagen), with expression of the proteins induced by the addition of 1 mm IPTG to the culture media. Cells were harvested by centrifugation and cell pellets were frozen at −20 °C for storage prior to protein purification.

Cell pellets were thawed on ice, and 25 mL of cells expressing each subunit were resuspended together in 250 mL 100 mm Tris pH 8.0, 150 mm NaCl, 1 mm EDTA (Buffer W, IBA Lifesciences, Göttingen, Germany). Cells were lysed using an Emulsiflex cell disrupter (Avestin, Mannheim, Germany) and the complex was purified using high‐capacity Strep resin (IBA Lifesciences), according to the manufacturer's instructions. This was followed by concentration and size‐exclusion chromatography using a Superose 6 10/300 column (GE Healthcare). The protein eluted from the column with a retention volume consistent with the expected size of the full AccA3–AccD6 dodecameric complex (~ 700 kDa). SDS/PAGE analysis of the eluted sample verified the presence of both AccA3 and AccD6 proteins. The collected sample was concentrated, buffer exchanged into 10 mm HEPES pH 7.0, 150 mm NaCl, and used for sitting drop crystallization experiments (0.5 μL drop size with 1 : 1 protein:mother liquor ratio).

Crystals grew after a period of several months [0.1 m Bis‐Tris pH 6.5, 25% poly(ethylene glycol) (PEG) 3350]. They were found to diffract to high resolution and data were collected without further crystal optimization. Diffraction data were collected at 100 K at Beamline X06SA (PXI), at the Swiss Light Source (Villigen, Switzerland).

Unit cell dimensions suggested that the crystals were unlikely to contain the entire AccA3:AccD6 complex, and the unit cell dimensions did not correspond to the previously solved AccD6 structures 24. Molecular replacement was performed using balbes 27 with AccA3 as the template sequence, and a structural solution was identified with a homodimer of AccA3 in the asymmetric unit, and no evidence of the AccD6 subunit.

The structural model was built initially using ARP/warp 28 followed by manual building using coot 29, and underwent several rounds of refinement (REFMAC5 and Phenix Refine) 30, 31 and rebuilding until a final model was obtained, a member of the ATP GRASP superfold, as expected from sequence analysis. In one monomer the GRASP A, B, and C domains were possible to build from the electron density. The other monomer had clear density for the A and C domains, and while it contained density appearing to correspond to a beta‐stranded core in the expected position for the B domain, this density could not be reliably built into or refined, suggesting that this domain may exist in several conformations in the crystallized form. The C‐terminal 150 residues of AccA3, expected to form an additional domain involved in the interaction with AccD proteins, were not located in the electron density. Coordinates and structure factors have been deposited in the protein data bank 32 PDB:5MLK.

Sequence searching, retrieval and alignments were performed using the programs blast 33, clustalw 34, and BOXSHADE within the SDSC Biology Workbench 35.

## Results and discussion

Crystals of MTb AccA3 (Rv3285) diffracted to beyond 2‐Å resolution, the molecular model was built and refined to 1.94 Å (Table 1).

**Table 1:** Data collection and refinement statistics

PDB id	5MLK
Data collection	
Wavelength (Å)	0.97
Resolution range (Å)	45.41–1.94 (2.06–1.94)
Space group	P2_1_2_1_2_1_
Unit cell a; b; c (Å)	77.9; 85.2; 148.1
Total reflections	957 449 (73 716)
Unique reflections	73 715 (11 692)
Multiplicity	13 (6.3)
Completeness (%)	99.8 (99.1)
Mean I/sigma (I)	19.01 (2.9)
R_merge_	0.12 (1.02)
CC_1/2_	0.999 (0.862)
Wilson B‐factor	26.4
Refinement	
R‐factor	0.189
R‐free	0.220
Number of atoms	6780
Protein	6369
Water	411
Protein residues	835
RMS (bonds; Å)	0.007
RMS (angles, °)	1.08
Ramachandran favored (%)	97
Ramachandran outliers (%)	0
Clashscore	4.28
Average B‐factor (Å^2^)	23.20
Protein (Å^2^)	23.10
Solvent (Å^2^)	24.20

Model validation statistics were calculated using the wwPDB OneDep deposition server service.

### Overall structure and B‐domain dynamics

**Figure 1:**
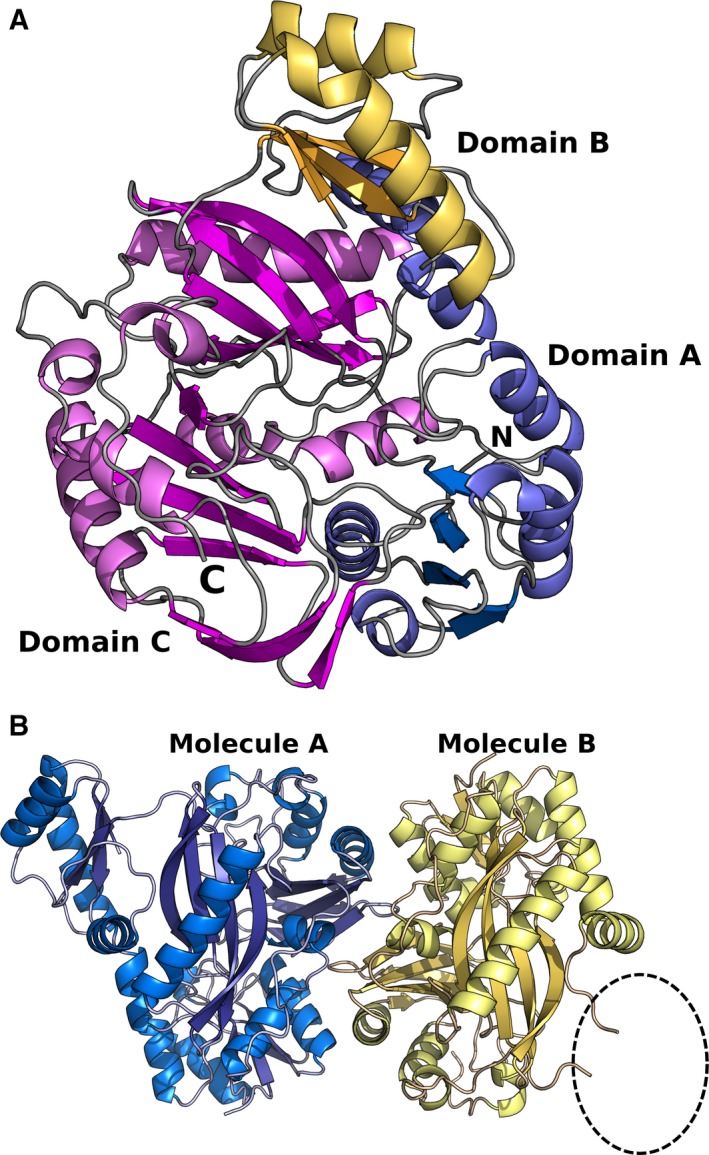
(A) Overall monomer fold of MTb AccA3 with domains colored in A: blue, B: yellow, and C: magenta. (B) Dimeric arrangement in the asymmetric unit. The dashed oval indicates the lack of an ordered structure for domain B of Molecule B.

*Mycobacterium tuberculosis* AccA3 adopts the ATP‐grasp superfamily fold (Fig. 1A), and crystallized as a dimer in the asymmetric unit (Fig. 1B), despite the full AccA3‐AccD6 dodecameric complex being used for crystallization. The dimerization interface has an area of ~ 1200 Å^2^ per monomer, burying some 8% of the total surface area of the dimer as calculated by PISA 36. Interestingly, the PISA analysis of the dimer interface does not suggest the formation of stable quaternary structures in solution. However, the dimer interaction architecture is conserved between AccA3 and previously determined structures of biotin‐dependent carboxylases 37, 38, 39, 40. Among these there are also examples where this interaction is predicted to be stable in solution 38. Together, this suggests that the observed dimer interface is not solely a result of crystal packing but biochemically relevant. The lack of a predicted stable dimerization interface is likely a reflection of the fact that AccA, and related proteins, need to be able to form different multimeric states and contacts in the homodimer and the AccA–AccD heteromultimer 41, 42. The multimer interaction interfaces must thus be sufficiently dynamic to allow release and rearrangement into other multimeric interaction architectures.

**Figure 2:**
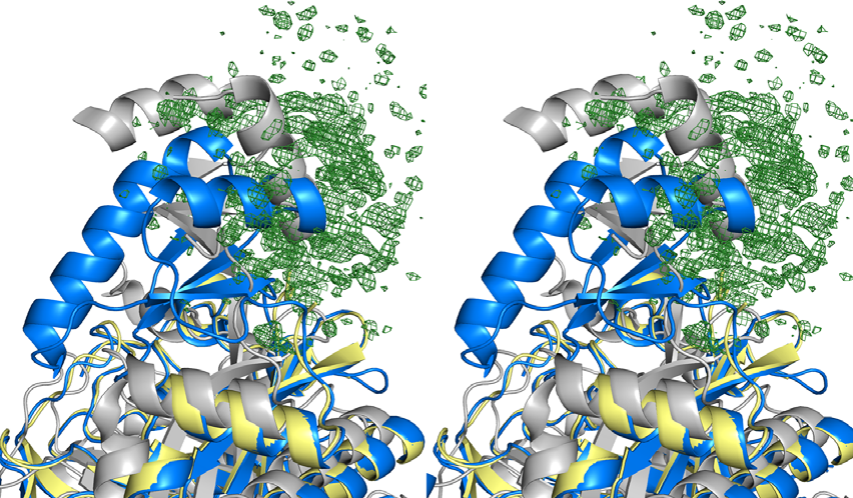
Stereo figure. Superposition of MTb AccA3, subunit A (blue) and subunit B (yellow) with the defined ‘open’ state conformation observed in nucleotide unbound BDC from *Escherichia coli* (gray) (PDB:1BNC) 43. Positive difference density [F_obs_ − F_calc_ contoured at 2.0 σ (0.21 e^−^·Å^−3^)] associated with subunit B of MTb AccA3 is displayed in green.

The protein crystallized in two different conformational states in the different subunits. In subunit A the protein chain could be traced from residues 10–465 with the exception of the glycine‐rich T‐loop connecting β‐strands two and three of the B‐domain comprising residues 173–177 (GGGKG). The T‐loop is a conserved feature and commonly observed to be disordered in the absence of bound nucleotide but orders upon nucleotide binding by interactions with the nucleotide phosphate groups 26, 40. The C‐terminal domain of AccA3, expected to mediate the interaction with the AccD subunit 41, 42, was not located in the electron density for any of the monomers. In protomer B, the entire B‐domain (residues 143–211) also appears structurally dynamic. There is a region of positive difference density significantly above background in the vicinity where the B‐domain is located in protomer B (Fig. 2). Trying to model this density, however, results in a poor fit and negative difference density around the modeled residues.

Previous structures have shown defined ‘open’ and ‘closed’ states of the B‐domain (Fig. 2) 40, 43. In addition, the biotin carboxylase domain of pyruvate carboxylase from *Bacillus thermodenitrificans* displays what appears to be an intermediate, but defined, conformation 44. In the current structure, however, while protomer A represents the previously observed ‘closed’ state, protomer B represent a different structural state where no conformation is present in high enough occupancy to be possible to reliably model. Based on the location of the segment of positive difference density relative to protomer B, it is, however, clear that the location of the B‐domain in the partially occupied structural state that gives rise to this density is not the same as either the previously described ‘closed’ or ‘open’ states. Rather, the density suggests an even more extended conformation of the B‐domain relative to the rest of the protein (Fig. 2). Together, the most likely interpretation of the combined structural data of biotin‐dependent carboxylases is that the B‐domain is dynamic over a continuum of conformations, or several defined conformations. This is of interest as it is presently unclear in what state the B‐domain resides in solution, in preparation for nucleotide binding 26. Calculations support a closed or a semiclosed state in solution 45, 46. The current structure suggests that, at least for MTb AccA3, there is no strongly preferred defined conformation for the B‐domain in the absence of substrate unless it, as in protomer A, is fixed in position by crystal contacts.

### Biotin‐ and ATP‐binding sites

**Figure 3:**
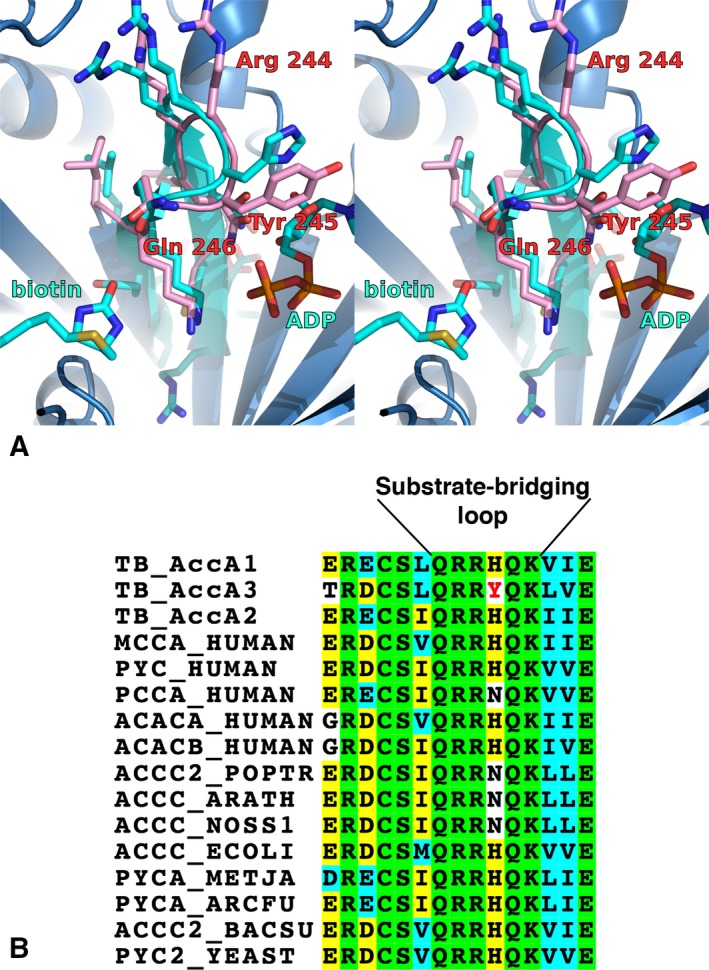
(A) Stereo figure. Structural model of biotin and ADP binding in MTb AccA3 based on the biotin and ADP‐bound *Escherichia coli* BDC (PDB:3G8C). Substrate‐bridging loop of MTb AccA3 rendered in pink and *E. coli* BDC in cyan. (B) Sequence alignment of a highly conserved region of the protein bridging the two binding sites and thus involved in binding of both substrates. Included are all MTb AccA homologs and all five human homologs in Swiss‐Prot, as well as a number of representative sequences from bacteria, eukaryotes and archaea (from Swiss‐Prot). Completely conserved residues with green background, the MTb AccA3‐specific tyrosine 245 is indicated in red.

The structure of MTb AccA3 displays well‐defined and deep substrate‐binding crevices, features that are suitable for inhibitor design and suggests that MTb AccA3 is indeed a druggable target. We were unable to obtain structures of ligand complexes of the MTb AccA3 via either soaking or cocrystallization. To gain insight into ligand binding and the binding sites we produced models of the biotin and ADP complexes based on the structures of *E. coli* BDC (PDB:3G8C, 44.9% identity over a 441 amino acid overlap, Cα rmsd 1.33 Å over 416 aligned residues; Fig 3A). The residues lining the substrate‐binding sites are, as expected, highly conserved. There is, however, one very interesting and rather drastic difference in the central part of the active site. Figure 3A shows the substrate‐binding sites MTb AccA3 compared to the biotin and ADP‐bound structure of *E. coli* BDC (PDB:3G8C) 40. The biotin‐ and ATP‐binding sites are bridged by a structurally conserved six‐residue loop structure linking two strands of the central β‐sheet. The residues of this loop interact with both substrates and are completely conserved between BDC homologs from all kingdoms of life, with the exception of one residue, which is conserved as a histidine in most organisms, with a possible substitution for asparagine in some cases, including one of the human homologs. MTb AccA3 is unique in this case, and also among the other MTb AccA proteins, where this position is replaced by a tyrosine (Fig. 3B). The residue in this position interacts directly with the bound nucleotide and is expected to influence the properties of the loop and the residues directly downstream which line the biotin‐binding site, for example, K247, the equivalent residue of K238 in the *E. coli* protein, shown to influence both the *K*_m_ for ATP as well as being involved in the carboxylation reaction 12, 47, 48. The substitution to tyrosine is likely to change the properties of both the nucleotide‐binding site and the active site‐bridging loop. It also suggests the possibility to design AccA3‐specific inhibitors, expected to be of great importance to avoid interaction with the human homologs of this protein.

In conclusion, the structure of MTb AccA3 suggests that the protein is a suitable target for drug design and reveals unique features of the active site that may be exploited to produce AccA3‐specific inhibitors to avoid interference with human metabolism.

## Author contributions

MB and MH designed the study, performed research, analyzed data, and wrote the paper.
